# Coartem^®^: the journey to the clinic

**DOI:** 10.1186/1475-2875-8-S1-S3

**Published:** 2009-10-12

**Authors:** Zulfiqarali G Premji

**Affiliations:** 1 Department of Parasitology/Medical Entomology, School of Public Health and Social Sciences, Muhimbili University College of Health Sciences, Box 65011, Dar-es-Salaam, Tanzania

## Abstract

Artemisinin, from which the artemether component of Coartem^®^(artemether/lumefantrine, AL) is derived, is obtained from the plant sweet wormwood (*Artemisia annua*) which has been used for over 2,000 years as a Chinese herbal remedy. Artemisinin was first identified by Chinese researchers as the active anti-malarial constituent of *A. annua *and its derivatives were found to be the most potent of all anti-malarial drugs. Artemether acts rapidly, reducing the infecting parasite biomass by approximately 10,000-fold per asexual life cycle. Lumefantrine, the other active constituent of AL, acts over a longer period to eliminate the residual 100-100,000 parasites that remain after artemether is cleared from the body and thus minimizes the risk of recrudescence. The two agents have different modes of action and act at different points in the parasite life cycle and show a synergistic action against *Plasmodium falciparum in vitro. *The combination of artemether and lumefantrine reduces the risk of resistance developing to either agent, and to date there are no reports of resistance to AL combined therapy in the malaria parasite that infects humans. Following a unique partnership agreement between Chinese authorities and Novartis, the manufacturer of AL, over 20 sponsored clinical studies have been undertaken in various malaria endemic regions and in travellers. These trials have involved more than 3,500 patients (including over 2,000 children), and led to identification of a six-dose, three-day regimen as the optimal dosing strategy for AL in uncomplicated falciparum malaria. AL has consistently shown 28-day polymerase chain (PCR)-corrected cure rates greater than 95% in the evaluable population, meeting WHO recommendations. More recently, Novartis and the Medicines for Malaria Venture have worked in partnership to develop Coartem^® ^Dispersible, a new formulation designed specifically to meet the specific needs of children with malaria. The dispersible tablets have shown similar high response rates to those observed with crushed standard tablets of AL. A partnership agreement between Novartis and WHO has seen over 250 million AL (Coartem^®^) treatments (75% for children) being distributed to malaria patients in developing countries without profit, supported by training programmes and educational resources.

## Background

Since it first received international licensing approval in 1999, Coartem^® ^(artemether/lumefantrine, AL) has become a mainstay of malaria treatment worldwide and is currently registered for use in almost 90 countries worldwide. Since 2002 it has been included in the WHO Model List of Essential Medicines, an index of priority drugs that guides purchasing decisions by United Nations agencies and many developing countries, and in 2007 it was added to the WHO Model List of Essential Medicines for Children. In 2004, Coartem^® ^became the first fixed-dose artemisinin combination therapy to be pre-qualified by the WHO, and received approval from the Food and Drug Administration in the US in April 2009.

## The discovery of artemisinin

The rapid-acting component of AL, artemether, is a derivative of artemisinin, a potent anti-malarial agent that is effective against multi-drug resistant parasites [1]. Artemisinin is obtained from the Chinese herb sweet wormwood (*Artemisia annua*). The first description of the use of sweet wormwood dates from the year 168 BC, when the plant was mentioned in a tomb of a member of the Han dynasty for the treatment of 52 diseases; in the year 1086 it was recommended in a Chinese compendium of medicines for the management of fevers and chills [2]. During the Vietnam War, the Chinese government systematically searched for an anti-malarial treatment from traditional Chinese medicine to support the Vietnamese army, and in 1972 artemisinin was identified as the active anti-malarial agent in *A. annua *[2].

**Figure 1:**
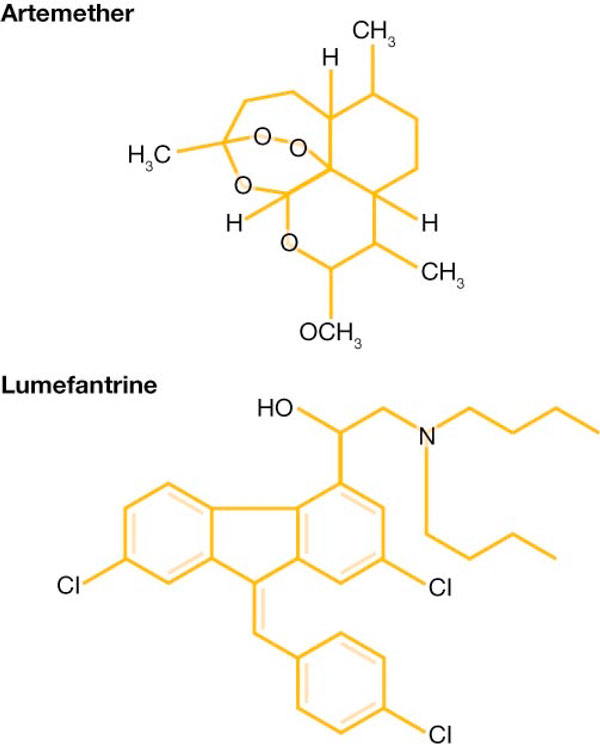
**Chemical structures of artemether and lumefantrine**.

Artemether (Figure 1) and other artemisinin derivatives have a broader spectrum of activity than other anti-malarial drugs, extending from the young ring stage of parasite development through to the early schizont [3]. Artemisinin derivatives proved to be the most potent of all anti-malarial drugs, reducing the infecting parasite biomass by approximately 10,000-fold per asexual life cycle i.e. every two days [4]. Importantly, artemisinin derivatives also markedly reduce gametocyte carriage, thereby limiting malaria transmission [1,5]. Several large-scale studies have demonstrated excellent gametocyte clearance [6-9]; indeed, in some trials no patient had gametocytes present two weeks after the first dose of AL was administered [6-8]. Artemisinin-based treatment has shown significantly greater gametocidal activity compared with other agents [10,11] and the widespread introduction of artemisinin-based therapy is believed to have contributed to reduced rates of malaria in sub-Saharan Africa.

## Advantages of the artemether/lumefantrine combination

Despite the potency of artemether, 100-100,000 residual parasites remain when the drug is used alone for a three-day treatment course, and as a result up to 10% of patients experience recrudescence [4,12]. It was recognized that combination treatment which eliminated the final parasites would be advantageous. The anti-malarial agent lumefantrine (Figure 1), which was originally synthesized by the Academy of Military Medical Sciences in Beijing [2], was identified by researchers at the Academy as a promising agent for combination with artemisinin. The two agents have different modes of action and act at different points in the parasite life cycle [1,3]. Artemisinin derivatives, such as artemether, have multiple mechanisms of action, including interference with parasite transport proteins, disruption of parasite mitochondrial function, modulation of host immune function and inhibition of angiogenesis [13]. Lumefantrine prevents the detoxification of haem, such that toxic haem and free radicals induce parasite death [1]. *In vitro*, artemether and lumefantrine show a synergistic action against *P. falciparum *[14]. Additionally, the differing pharmacokinetics of the two agents offer an advantage for combination therapy. Artemether and its main active metabolite dihydroartemisinin (DHA) are extremely potent anti-malarials that reach peak concentration quickly (at approximately two hours [3]) and are eliminated rapidly (elimination half-life 1-3 hours) [1,3]. Lumefantrine is absorbed and eliminated more slowly than artemether, with a terminal elimination half-life of 4-5 days [1]. As described in the subsequent chapter '*Understanding the Pharmacokinetics of Coartem*^®^', these characteristics work synergistically: the rapidly-absorbed artemether and DHA achieve a fast reduction in parasite biomass and prompt symptomatic improvement, while the lumefantrine concentrations that persist in the blood after the three-day treatment course eliminate any remaining parasites to prevent recrudescence [3].

The combination of artemether and lumefantrine would also be expected to restrict the development of drug resistance to either agent. Combination therapy is now widely accepted as the way forward to slow the rapid emergence and spread of resistance to malaria, and to increase the useful therapeutic life of anti-malarial drugs. Indeed, in 2006 WHO called for manufacturers to stop selling monotherapy artemisinin treatments in an attempt to prevent malaria parasites developing resistance to the drug [15]. Concomitant administration of artemether and lumefantrine reduces the chance of survival by a resistant organism, since their different sites of action mean that mutations resistant to both agents would be required for the organism to survive [4]. This advantage only applies to drug combinations in which the organism is not already resistant to one or more of the drugs, which was the case for both artemether and lumefantrine. To date, there are only two recorded cases of resistance to artemisinin derivatives, both reported in patients receiving artesunate monotherapy in Western Cambodia [16] a region that has seen approximately 30 years of artemisinin use in various doses and formulations and perhaps the greatest exposure to artemisinin of anywhere in the world. While parasites from these two patients showed reduced susceptibility *in vitro*, the authors commented that artemisinin resistance does not seem to be a widespread epidemiologic phenomenon and in a subsequent publication by White *et al *pointed out that malaria parasites from Western Cambodia are no more resistant *in vitro *than parasites from other regions [17]. No cases of resistance have been reported for patients receiving artemether/lumefantrine together, in AL. Lumefantrine, unlike other companion drugs used in artemisinin-based therapies, was never used as monotherapy prior to combination with artemether in AL, and has thus not been exposed to the risk of resistance that this entails. It is known that long elimination times facilitate the spread of resistant parasites once a mutation arises [5], because this increases the chance of a second, unrelated infection while drug concentrations are low and the likelihood of within-patient selective pressure when an infection is not eradicated from the body [4]. The short elimination half-life of artemether means that it has a low potential for selection of resistant parasites [1]. While the elimination half-life of lumefantrine is longer (4-5 days), it is markedly shorter than the half-lives of agents such as mefloquine (2-3 weeks) and choloquine (1-2 months), against which significant resistance has emerged [5], and piperaquine, for which drug resistance has also been reported and which has an elimination half-life of approximately 17 days in children with malaria [18].

## The clinical development programme

During the 1980s and 1990s, researchers at the Beijing Academy of Military Medical Sciences investigated combined administration of artemether with lumefantrine. From 1994 onwards, this work was undertaken in collaboration with Novartis, the manufacturer of AL (Coartem^®^), in the first joint development programme of its type in Chinese history. Preclinical studies indicated that a 1:6 fixed combination of artemether and lumefantrine was well tolerated even at doses 10 times higher than those used in subsequent clinical trials [3], and based on the results from clinical trials in several hundred patients it was concluded that a 1:6 weight/weight ratio was the optimum combination [3]. These early studies showed that the AL combination was an effective and well-tolerated treatment for uncomplicated falciparum malaria [19-22].

**Figure 2:**
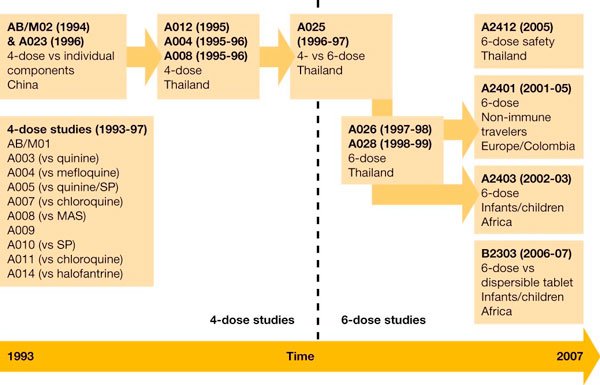
**Overview of the clinical development programme for AL**.

Over 20 clinical studies of AL supported by Novartis have now been undertaken in various malaria endemic regions and in travellers (Figure 2). In total, 3,599 patients, including 1,572 adults and 2,027 children, have taken part in these trials. Additionally, approximately 45 independent clinical trials have been published in the scientific literature. These have each used a twice-daily regimen that maintained the blood concentration of artemether above the minimum effective concentration [1], to ensure that the infecting parasites are all exposed to high levels of artemether when they are in the middle third of their lifecycle - the point at which they are most susceptible to anti-malarial agents [23]. Early trials using a four-dose regimen of AL, with a twice-daily regimen that maintained the blood concentration of artemether above the minimum effective concentration [1], showed excellent parasite and fever clearance with high cure rates [19,21,22]. Subsequently, however, a randomized, double-blind study was undertaken in Thailand, in which 359 adults and children with uncomplicated falciparum malaria received either a four-dose or six-dose twice-daily regimen of AL [24]. While parasite and fever clearance time were similar with either a four- or six-dose schedule, six doses resulted in a 28-day PCR-corrected cure rate of >95% in evaluable patients, significantly higher than that seen in patients given a four-dose course (83.3%, p < 0.001). Tolerability was unaffected by use of a six-dose regimen. On this basis, studies have since employed the now-standard six-dose regimen, and subsequent randomized trials in the same geographic region [25,26] and elsewhere [27] have confirmed PCR-corrected cure rates above 95% among evaluable patients [19,20], meeting the WHO target response rate [5].

A discussion of more recent trial results is included in '*The clinical efficacy of artemether/lumefantrine (Coartem^®^)*' in this supplement [28].

The evaluation of AL in specific clinical settings is ongoing. One prospective, randomized trial involving over 200 patients is currently underway in Columbia with the aim of confirming that AL does not affect auditory function, while an observational pregnancy study is being conducted at four antenatal clinics in Zambia to assess outcomes in mothers and newborns after use of AL therapy during the second or third trimester. In a rural area of southern Tanzania, an epidemiological study is being carried out to investigate the impact of the introduction of AL as a national treatment policy on all-cause mortality, malaria transmission, safety and adherence in infants and children younger than five years old.

## Development of Coartem^® ^Dispersible

Until recently, there was no WHO-endorsed paediatric formulation for the treatment of malaria. Working in partnership, Novartis and the Medicines for Malaria Venture (MMV) have developed a formulation of AL tailored to the needs of children with *P. falciparum *malaria. The previous standard of care, crushed standard tablets of AL, achieved 28-day PCR-corrected cure rates of >95% among evaluable patients [6,25,29], but was inconvenient for caregivers and difficult to administer to sick children, while the crushing process risked loss of drug and the bitter taste meant that children might spit it out. The new dispersible formulation received its first approvals for use in 2008, followed by further approvals including Switzerland in 2009, and was pre-qualified by the WHO in 2009. (See '*Dispersible formulation of artemether/lumefantrine: specifically developed for infants and young children' *in this supplement for more details) [30].

## Distribution of AL to malaria endemic countries

Following a ten-year agreement with WHO in 2001, the manufacturer Novartis made AL (Coartem^®^) available without profit for distribution through WHO in malaria-endemic developing countries. More than 250 million treatments have been provided through this without-profit arrangement, 75% of which were for children. In 2008 alone, 74 million treatments were distributed. This unexpected demand for AL has necessitated a dramatic growth in commercial farming of high-quality *A. annua *plants in Kenya and China, as well as the establishment of extensive new production facilities in China, Africa and the US. In addition, the widespread adoption of AL has been supported by a series of training programmes and educational materials for health professionals and patients or carers, to help ensure optimal prescribing and adherence to the AL regimen.

### Home-based management using AL

One vital area of research is the development of strategies for the use of home-based malaria management using AL [31-34] in order to address the problem of poor access to health centres particularly in poor, rural areas [35]. There are obstacles to be overcome, including misdiagnosis at home on the basis of symptoms alone, over-prescribing with risk of resistance, and achieving adequate availability of AL for home treatment [36]. A study by Dunyo *et al *has shown, however, that the accuracy of presumptive diagnoses by carers and health centres was similar when confirmed by parasitological analysis, and the diagnosis was typically made three days earlier at home, helping to prevent complications that arise from persistence of symptoms and progression to high levels of parasitaemia [37]. Intervention studies in which AL was made available locally for home management have shown encouraging results, providing prompt treatment [31,34], with good adherence to the correct prescribing regimen [32], while reducing the case burden at health facilities [33]. The risk of over-prescription of AL can, however, can be an issue with carer administration [34], and distribution through trained community health workers - if available - may help to ensure appropriate prescribing, especially if the community team is equipped with rapid diagnostic testing (See *'Impact of large-scale deployment of artemether/lumefantrine on the malaria disease burden in Africa: case studies of South Africa, Zambia and Ethiopia' *for more details [38].)

## Conclusion

AL has had a remarkable journey to the clinic, from an ancient Chinese herbal remedy to a modern medicine that meets the most stringent drug approval criteria, and over 250 million Coartem^® ^treatments have now been delivered. A long series of clinical trials has assessed the efficacy, safety and pharmacokinetics of AL - and more recently the dispersible formulation of AL - in a wide range of geographic regions and patient types, achieving cure rates higher than 95% among evaluable patients, with good tolerability. Ongoing investigations continue to ensure optimal safety and to identify the best routes for prescribing and distribution of AL, while AL continues to be provided in developing countries through non-profit production from Novartis with distribution via WHO.

## Competing interests

The author would like to acknowledge that Novartis Pharma AG sponsored this supplement. However, the author does not work for, or represent in any way, Novartis Pharma AG.

## Authors' contributions

The author met International Committee of Medical Journal Editors criteria for authorship.
